# Alcohol Use Among Cuban-Americans, Mexican-Americans, and Puerto Ricans

**Published:** 1998

**Authors:** Whitney M. Randolph, Christine Stroup-Benham, Sandra A. Black, Kyriakos S. Markides

**Affiliations:** Whitney M. Randolph is a doctoral candidate, Christine Stroup-Benham, PhD., is an assistant professor, and Kyriakos S. Markides, Ph.D., is a professor in Preventive Medicine and Community Health and Sandra A. Black, Ph.D., is an assistant professor in the Center of Aging at The University of Texas Medical Branch, Galveston, Texas

**Keywords:** Hispanic, amount of AOD use, AOD use frequency, alcohol consumption, gender differences, acculturation, West Indies and Bermuda, Mexico, Central American, immigrant, ethnic differences, racial differences, morbidity, AODR (alcohol and other drug related) morbidity, literature review

## Abstract

Studies of alcohol consumption among Hispanics in the United States show different patterns based on gender, country of origin, and economic status. The literature shows a lower frequency but higher volume of consumption among Mexican-American and Puerto Rican males than among non-Hispanic white males. Cuban-American males have a pattern of relatively moderate consumption that resembles that of non-Hispanic whites. Women from Mexican-American, Cuban-American, and Puerto Rican populations have low alcohol consumption rates, which appear to increase with the level of acculturation. Existing data are old, however, and new studies are needed to update information on alcohol consumption patterns of the largest Hispanic subgroups in the United States as well as the drinking habits of the growing numbers of Hispanics from places other than Mexico, Puerto Rico, and Cuba.

Hispanic-Americans are one of the fastest growing minority groups in the United States, numbering more than 28 million and making up about 11 percent of the U.S. population (U.S. Bureau of the Census 1997). Approximately 60 percent of all Hispanics in the United States are of Mexican origin, 15 percent are of Puerto Rican origin, and 5 percent are of Cuban origin; the remaining 20 percent is composed of people with origins in other Spanish-speaking nations of the Caribbean, Central America, and South America (U.S. Bureau of the Census 1997). In recent years, increased interest has developed in understanding more about this population’s health status and health behaviors. This article provides a brief overview of what is known about alcohol consumption among Hispanic-Americans, including information on the influence of such factors as country of origin, gender, and level of acculturation. The article also examines Hispanics’ patterns of alcohol consumption (i.e., frequency and volume), associated problems, and the ways in which their drinking patterns compare with those of non-Hispanic whites and African-Americans (see table 1, p. 266).

## Alcohol Consumption Among Hispanic-American Men

Early research reviewed by Caetano (1983) and Neff (1986) indicated that Hispanic-American men appeared to drink less frequently but in higher quantities than non-Hispanic white and African-American men, a pattern sometimes referred to as binge, or “fiesta,” drinking (table 1). Data from the 1982–1984 Hispanic Health and Nutrition Examination Survey (HHANES), which included Mexican-Americans in the Southwest, Puerto Ricans in the New York City area, and Cuban-Americans in Dade County (Miami), Florida, confirmed this pattern among Mexican-American and Puerto Rican males, but not among Cuban-Americans (Lee et al. 1997).

A number of studies within specific Hispanic populations have found that differences in alcohol consumption among males exist based on Hispanic origin (see table 2, p. 267). A 1984 survey of drinking practices and alcohol consumption conducted by the Alcohol Research Group (ARG) found that although Mexican-American men had higher rates of abstention than other Hispanic men, they also had the highest rate of frequent heavy drinking, defined as consuming five or more drinks^1^ in one sitting at least once per week (Caetano 1988). In the ARG study, 54 percent of the Mexican-American men reported heavy drinking (as defined above) at least once per year and as often as once per week, whereas 28 percent of the Puerto Rican men and only 8 percent of the Cuban-American men reported drinking patterns consisting of the same high volume and frequency (Caetano 1988). Despite limited numbers of Cuban-Americans and Puerto Ricans in the study, the data indicated that Mexican-American men were more likely to drink large quantities of alcohol in any given drinking session than were Puerto Rican and Cuban men (Caetano 1988). Other studies also have observed Mexican-American men to drink heavily and become frequently intoxicated (Gilbert and Cervantes 1986). Compared with other Hispanic populations, alcohol consumption among Cuban-American males appears to be more frequent but more moderate in volume, much like the pattern for non-Hispanic whites (Black and Markides 1994). Recent Cuban immigrants, however, appear to have patterns of alcohol consumption and alcohol problems that resemble those of Mexican-Americans and Puerto Ricans. Those patterns may result from recent Cuban immigrants’ having a socioeconomic status similar to that of Mexican-Americans and Puerto Ricans. In contrast, Cubans who immigrated to the United States in the 1960s have a socioeconomic status comparable to that of the general U.S. population (Black and Markides 1994).

One analysis of data from the HHANES found that Mexican-American and Puerto Rican men were much more likely than Cuban-American men to report that they had experienced a period in their lives when they considered themselves to be heavy drinkers. The survey also found that those who considered themselves to have been heavy drinkers in the past were more likely to be current heavy drinkers (Lee et al. 1997).

In addition to differences across national origin groups, Hispanic males also appear to have drinking patterns that differ by age. Using the HHANES, Black and Markides (1994) examined life-course drinking patterns among Mexican-American, Cuban-American, and mainland Puerto Rican men ages 25 to 74. They found that across all three national origin groups, abstinence was highest in the older age groups (i.e., ages 45 to 74), with the highest proportion (40.4 percent) among Cuban-American males ages 45 to 54. The largest proportion of current drinkers for all ages and national origins was found among Mexican-Americans ages 25 to 34, of whom 84.6 percent could be classified as current drinkers. Former drinkers were most numerous among men ages 55 to 74, regardless of their national origin. Cuban-American males had the most conservative pattern of alcohol consumption across all age groups in the study. Other research also has suggested that the pattern of heavy drinking by Mexican-American and Puerto Rican men continues into middle age, later than has been found among non-Hispanic white and Cuban-American men (Gilbert and Cervantes 1986; Markides et al. 1990).

## Effects of Acculturation

Among researchers, a lively interest has grown in examining the influence of various measures of acculturation on the alcohol consumption of Hispanic populations. Typically, those measures include items capturing language use and preference, ethnic identification, and nativity of both the respondent and his or her parents. Thus, the more acculturated a person is, the more likely he or she is to speak or prefer to speak English over Spanish, to identify as American rather than of Hispanic origin, and to have been born in the United States to U.S.-born parents.

The effects of acculturation on alcohol consumption among Hispanic males have been studied in many different Hispanic groups, but because it is a complex issue, results have varied. One study suggested that less acculturated middle-aged Mexican-American males drink more heavily than Mexican-American males who are more acculturated, suggesting a possible acculturative stress effect^2^ (Cervantes et al. 1990/1991). The effects of acculturation also appear to vary by age across Hispanic groups, with increased acculturation related to less abstention in older men and to frequent heavy drinking in younger men (Caetano 1987). Overall, acculturation seems to have less of an effect on men than on women. Again, this area of research is a complex one, and findings are contradictory. Clearly, age, gender, and many other factors ultimately influence the behavioral and psychological response to moving from one culture to another.

## Alcohol Consumption Among Hispanic-American Women

Research on Hispanic drinking has shown that despite variation in alcohol consumption among different groups, heavy drinking and drunkenness is primarily a male activity, whereas abstention and infrequent light drinking is the common pattern for women (Caetano 1988; Canino 1994; Gilbert and Cervantes 1986). Women in all Hispanic groups consume significantly less alcohol than do their male counterparts and tend to abstain from alcohol completely or consume it in small quantities. Moreover, Hispanic women consume alcohol less frequently and at lower volumes than do non-Hispanic white and African-American women. For example, whereas more than 85 percent of non-Hispanic white women have reported consuming alcohol in their lifetime, fewer than 63 percent of Hispanic women have ever consumed alcohol (Cervantes et al. 1990/1991). Traditional Hispanic values do not sanction drinking by women, particularly drinking large quantities of alcohol (Canino 1994). This cultural norm may be responsible for Hispanic women’s drinking patterns.

Differences in Hispanic women’s drinking patterns do exist based on age, level of acculturation, and country of origin. Data from the HHANES, for example, showed that Mexican-American and Puerto Rican women consumed a higher volume of alcohol and more often reported heavy drinking^3^ than did the Cuban-American women, who drank the least of the three groups (Black and Markides 1993).

Data from the HHANES also suggested other factors that may influence the drinking patterns of Hispanic women. For instance, employed women tended to drink more frequently but less heavily than unemployed women. Women in poverty drank less frequently than women not in poverty but drank more per drinking episode. Mexican-American women who were married also drank less frequently and consumed smaller volumes of alcohol than did unmarried women, and women with higher levels of education were also more likely to drink (Black and Markides 1993; Markides et al. 1990). The relationships between drinking and sociodemographic factors were less clear among Puerto Rican and Cuban-American women, but higher levels of education also appeared to be associated with a greater probability of being a drinker in the two groups.

A number of studies have shown that a higher level of acculturation is related to greater alcohol consumption, especially in relatively young Hispanic women (Black and Markides 1993; Caetano 1987; Gilbert and Cervantes 1986). Those findings are consistent with the *acculturation model*, which predicts that alcohol consumption of Hispanic women reflects the extent to which they have adopted the drinking norms and practices of the larger society (Markides et al. 1990). Higher acculturation to U.S. society by younger women may mean less adherence to traditional cultural norms; thus, young, acculturated Hispanic-American women may ignore taboos about female alcohol consumption, and their alcohol consumption patterns may then become more like those of non-Hispanics. Some evidence indicates that the level of acculturation may not be associated with alcohol consumption among middle-aged Mexican-American women, whose volume of consumption appears instead to be positively influenced by poverty and marital disruption (Markides et al. 1990).

For women of all ages in all three major subgroups in the HHANES, acculturation was consistently correlated with both increased frequency of consumption and increased probability of being a drinker at all (Black and Markides 1993). Acculturation also was positively associated with the total number of drinks that Cuban-American and Mexican-American women consumed during the 4-week period before their interview and with the volume of alcohol that Mexican-American women consumed per occasion during that time (Black and Markides 1993).

## Health-Related Consequences of Heavy Drinking

Chronic heavy drinking can result in alcohol-related morbidity and mortality. Both U.S. and native Mexican populations have a higher rate of alcohol-related mortality from cirrhosis of the liver than does the general U.S. population, and Mexican-Americans may be disproportionately represented in alcohol-related violence (Gilbert and Cervantes 1986). A recent study of whites, African-Americans, and Hispanics showed an increased prevalence of alcohol problems (based on answers to 14 questions about such topics as physical morbidity, accidents, and interpersonal conflicts) among Hispanic males from 1984 to 1995, but it showed no change among African-Americans and whites (Caetano and Clark 1998).

National data have shown that Hispanics of both genders have higher rates of death from cirrhosis and liver disease than do non-Hispanics (Sorlie et al. 1993). Although this difference may appear to be easier to explain in men, women’s low alcohol consumption levels suggest that factors other than alcohol may be responsible for those high death rates for Hispanic women and, possibly, for men. For example, hepatitis C may be a contributing factor to cirrhosis rates among Hispanics, because Hispanic ethnicity has been found to be an independent risk factor for exposure to the hepatitis C virus (Murphy et al. 1996). Like the HIV virus, hepatitis C transmission is based on behavior; the higher incidence of hepatitis C is likely related to an underlying behavior, which has not been identified. Other factors, such as social support, diet, or genetics, may be responsible for the rates of liver disease.

## Summary

Although alcohol consumption varies greatly on an individual level in all cultural groups, certain macro-level trends can be observed in the drinking patterns of Hispanic-Americans. Consumption appears to vary by gender, age, level of acculturation, and national origin. The Cuban-Americans who came to the United States in the 1960s tended to have a fairly high socioeconomic status compared with other Hispanic groups and typically display more moderate drinking patterns than do Mexican-Americans and Puerto Ricans. Specifically, studies of Mexican-American men reveal that many of those who do drink engage in low-frequency, high-volume binge drinking. Young Hispanic men have been found to have the highest level of alcohol consumption, whereas the oldest cohorts of men have very low levels of consumption. Among Hispanic-Americans, Cuban-American women appear to drink the least amount of alcohol. In addition, younger and more acculturated women drink more in quantity and frequency than do less acculturated or older women in all three Hispanic groups. Greater assimilation into American society by Hispanics may ultimately change the taboos against alcohol consumption by women and may encourage Hispanic men to become more frequent but lower quantity drinkers.

Data about individual subgroups may now be dated, because data from large samples, such as the HHANES, are approximately 15 years old and may be geographically skewed. More current regional studies, however, tap only into specific geographic areas of Hispanics that are not representative of all U.S. geographic areas. Large representative studies of the various Hispanic subgroups are needed to update information on the alcohol consumption patterns of those populations. Especially needed are studies of the growing numbers of Hispanics from places other than Mexico, Puerto Rico, and Cuba.

## Figures and Tables

**Table 1:** Drinking Patterns of U.S. Drinkers by Gender, Ethnicity, Income, and Education (1990)

	Drinking Pattern (% of respondents)		
	
Respondent Characteristic	Current	Weekly	Heavy*
**Gender**			
Female (1,189)**	59.4	18.8	1.4
Male (869)	71.2	40.0	6.5
**Ethnicity**			
Black (261)	61.6	25.8	3.5
White (1570)	65.9	30.2	3.5
Hispanic (150)	66.6	26.5	8.9
Other (77)	57.0	21.6	1.4
**Income**			
> Median	73.8	31.7	2.5
< Median	56.1	25.5	4.9
**Education**			
Less than high school	50.4	23.5	6.3
High school	66.3	26.4	3.8
Some college	70.2	30.6	3.1
College	75.4	39.8	1.8

* Heavy drinkers are those who reported having five or more drinks on one occasion at least once per week during the previous year.

** Numbers in parentheses indicate the number of respondents in each category.

SOURCE: Adapted from National Institute on Alcohol Abuse and Alcoholism 1997.

**Table 2:** Drinking Patterns by Type of Hispanic Origin and Gender

		Type of Hispanic Origin (% of repondents)			
		
Drinking Pattern	All Hispanic	Mexican	Puerto Rican	Cuban	Other Hispanic Origin
	(845f*/604m*)	(539f/410m)	(141f/78m)	(50f/45m)	(104f/66m)
**Frequent Heavy**					
Female	3	2	2	< 0.5	4
Male	17	18	16	5	15
**Moderate**					
Female	3	2	6	4	5
Male	11	6	7	10	32
**Light**					
Female	9	10	6	11	6
Male	15	4	41	38	21
**Abstainer**					
Female	47	46	33	42	69
Male	22	27	19	12	9

* Numbers in parentheses indicate the number of female (f) and male (m) respondents from each type of Hispanic origin.

NOTE: Frequent heavy = drinks five or more drinks at one sitting at least once per week; moderate = drinks at least once per week but never more than five drinks at one sitting; light = drinks one to three times per month but never more than five drinks at one sitting; and abstainer = drinks less than once per year or has never consumed alcohol.

SOURCES: Caetano 1988, 1989.

## Footnotes

1 Researchers generally define a “standard drink” as one 12-ounce beer or wine cooler, one 5-ounce glass of wine, or 1.5 ounces of 80-proof distilled spirits.

2 An acculturative stress effect is a situation in which the difficulties of adapting to a new culture induce various behavioral and psychological responses, which may include drinking, depression and other psychological disorders, and interpersonal problems.

3 For women, the study defined heavy drinking as the consumption of four or more drinks per occasion.
